# Cancer in Pregnancy: Therapeutic Decisions at the Intersection of Two Lives

**DOI:** 10.3390/diagnostics16071070

**Published:** 2026-04-02

**Authors:** Antônio Braga, Jorge de Rezende-Filho, Maurício Magalhães, Sue Yazaki Sun, Andreia Cristina de Melo, Gustavo Yano Callado, Fernanda da Costa Negraes, Maria Vitória Moura Fajardo, Susana Cristina Aidé Viviani Fialho, Edward Araujo Júnior, Glória Calagna

**Affiliations:** 1 Department of Gynecology and Obstetrics, School of Medicine, Maternity School, Federal University of Rio de Janeiro, Rio de Janeiro 22240-003, RJ, Brazil; bragamed@yahoo.com.br (A.B.); rezendef@me.ufrj.br (J.d.R.-F.); 2 Postgraduate Program in Medical Sciences, School of Medicine, Federal Fluminense University, Niterói 24070-090, RJ, Brazil; 3 Department of General and Specialized Surgery, School of Medicine and Surgery, Federal University of Rio de Janeiro, Rio de Janeiro 20270-004, RJ, Brazil; 4 Postgraduate Program in Applied Health Sciences, University of Vassouras, Vassouras 27700-000, RJ, Brazil; 5 National Academy of Medicine, Rio de Janeiro 20021-130, RJ, Brazil; mamcosta@yahoo.com; 6 Department of Obstetrics, Paulista School of Medicine, Federal University of São Paulo, São Paulo 04023-062, SP, Brazil; sueysun@gmail.com (S.Y.S.);; 7 Division of Clinical Research and Technological Development, Brazilian National Cancer Institute, Rio de Janeiro 20231-050, RJ, Brazil; andreia.melo@inca.gov.br; 8 Faculdade Israelita de Ciências da Saúde Albert Einstein, Hospital Israelita Albert Einstein, São Paulo 05652-900, SP, Brazil; gycallado@gmail.com; 9 Service of Gynecology and Obstetrics, Antônio Pedro University Hospital, Federal Fluminense University, Niterói 24070-090, RJ, Brazil; nandac.negraes@gmail.com (F.d.C.N.); mariavitoriafajardo06@gmail.com (M.V.M.F.); 10 Department of Maternal and Child Health, School of Medicine, Federal Fluminense University, Niterói 24070-090, RJ, Brazil; susanaaide@id.uff.br; 11 Discipline of Women’s Health, Municipal University of São Caetano do Sul, São Caetano do Sul 09521-160, SP, Brazil; 12 Villa Sofia Cervello Hospital, University of Palermo, 90100 Palermo, Italy

**Keywords:** cancer in pregnancy, gestational cancer, chemotherapy during pregnancy, obstetric oncology, placental metastasis, multidisciplinary care

## Abstract

**Background**: Cancer during pregnancy is a rare but increasingly encountered condition due to delayed childbearing and improved survival of women with cancer. The coexistence of malignancy and pregnancy poses complex diagnostic and therapeutic challenges, requiring careful balance between maternal prognosis and fetal safety. **Objective**: This review aims to summarize current evidence on the diagnosis and multidisciplinary management of cancer during pregnancy, focusing on the safe use of oncologic therapies, obstetric decision-making, and maternal and fetal outcomes. **Methods**: A narrative review was conducted based on literature identified in PubMed/MEDLINE, Scopus, and Web of Science from January 2000 to September 2025. Search terms included pregnancy-associated cancer, oncologic treatment during pregnancy, obstetric management, and fetal outcomes. Relevant clinical guidelines, registry data, and reference lists were also reviewed. **Results**: Breast cancer, cervical cancer, melanoma, hematologic malignancies, and gestational trophoblastic neoplasia represent the most frequently reported cancers during pregnancy. Evidence indicates that selected cancer treatments can be administered safely without compromising maternal prognosis. Chemotherapy after the first trimester is generally associated with acceptable fetal outcomes. Physiological changes in pregnancy may delay diagnosis and influence drug pharmacokinetics. Radiotherapy, targeted therapies, and immunotherapies remain limited because of potential fetal toxicity. Avoiding iatrogenic prematurity is a central principle of obstetric management. **Conclusions**: Cancer during pregnancy should no longer be considered an absolute therapeutic dilemma. With individualized multidisciplinary care, effective maternal treatment and favorable fetal outcomes are increasingly achievable.

## 1. Introduction

Cancer associated with pregnancy is defined as a malignancy diagnosed during gestation or within one year after delivery. Its most frequent types reflect the epidemiological profile of neoplasms affecting women of reproductive age, with breast and cervical cancers, melanoma, and hematologic malignancies being the most prevalent [1,2]. Studies indicate that the incidence of pregnancy-associated malignancies has increased over time [3]. This rise occurs in parallel with both the earlier onset of certain cancer types and, most notably, the contemporary trend toward delayed motherhood, which is related to multiple factors, including educational and professional goals, economic pressures, and transformations in social norms regarding marriage and family formation [4,5]. As a consequence of this postponement, an increase in maternal age at conception has been observed, a factor that significantly contributes to a higher risk of cancer development during pregnancy [3].

Cancer during pregnancy poses substantial challenges related to diagnosis, prognosis, and therapeutic management in this population. Physiological changes inherent to pregnancy may act as confounding factors, hindering early disease recognition. Moreover, although advances in imaging techniques have expanded diagnostic capabilities, some modalities remain contraindicated or restricted during pregnancy, potentially delaying timely diagnosis [2,4]. According to the integrative review conducted by Brito et al., there is evidence that pregnancy does not accelerate cancer progression; rather, poor prognosis is primarily associated with advanced tumor stage at diagnosis, reinforcing the importance of early detection for improving outcomes in these cases.

Furthermore, the psychological impact of cancer during pregnancy on women’s mental, emotional, social, and spiritual health should not be overlooked. Symptoms such as distress, persistent suffering, anxiety, and fear—frequently associated with an oncologic diagnosis—tend to be exacerbated during pregnancy, a period characterized by heightened emotional and psychological vulnerability [4,6]. This aspect is particularly relevant given the association between maternal exposure to stress during pregnancy and adverse outcomes, including preterm birth, low birth weight, and alterations or impairments in the child’s neurodevelopment [7,8].

For these reasons, optimal management of cancer during pregnancy requires a patient-centered, multidisciplinary approach grounded in ethical principles that prioritize shared decision-making. This approach should take into account the values of the pregnant woman and her family, her tolerance for treatment-related risk, and fetal viability according to gestational age. Critical decisions—such as the timing of surgical intervention, initiation or postponement of chemotherapy, delivery planning, and consideration of pregnancy termination—should be evaluated at relevant gestational milestones, with appropriate documentation and clear, open, and patient-centered communication [4]. Although clinical guidance has been provided by major international societies, including the European Society of Gynaecological Oncology (ESGO) [2], the American Society of Clinical Oncology (ASCO) [4], and the European Society for Medical Oncology (ESMO) [9], the field of cancer during pregnancy continues to evolve rapidly. The emergence of novel oncologic therapies and the expansion of large international collaborative registries have generated new data that increasingly inform clinical decision-making. These advances highlight the need for an updated and comprehensive synthesis of current evidence.

The motivation for this review is to provide an updated and comprehensive synthesis of the current evidence on cancer during pregnancy, integrating oncologic management, obstetric decision-making, and maternal–fetal outcomes. In contrast to many previous publications that focus on specific tumor types or individual therapeutic approaches, the present review aims to provide a multidisciplinary overview of the diagnosis and management of pregnancy-associated cancers. Given the clinical relevance and inherent challenges of managing cancer during pregnancy, the present study aims to synthesize current knowledge on the management of cancer during pregnancy by addressing four central questions: (1) What is the epidemiology and clinical presentation of the most common cancers during pregnancy? (2) How do the physiological adaptations of pregnancy influence cancer diagnosis and treatment? (3) What are the current evidence-based recommendations regarding chemotherapy, surgery, radiotherapy, and emerging targeted therapies? and (4) What are the obstetric, neonatal, and long-term outcomes for both mother and child?

## 2. Methods

This narrative review was based on a comprehensive literature search conducted in the PubMed/MEDLINE, Scopus, and Web of Science databases.

### 2.1. Search Strategy and Data Sources

The search strategy combined Medical Subject Headings (MeSH) terms and free-text keywords, including “pregnancy,” “cancer,” “malignancy,” “chemotherapy,” “radiotherapy,” “surgery,” “targeted therapy,” “immunotherapy,” “fetal outcomes,” “placental metastasis,” “obstetric management,” “long-term outcomes,” and “ethics.” Searches covered the period from January 2000 to September 2025.

Reference lists of relevant articles were manually screened to identify additional studies. Clinical practice guidelines from major learned societies, including the ESGO [2], the ASCO [4] and the ESMO [9], as well as reports from international collaborative registries, particularly the International Network on Cancer, Infertility and Pregnancy (INCIP) [8], were also reviewed.

**Figure 1:**
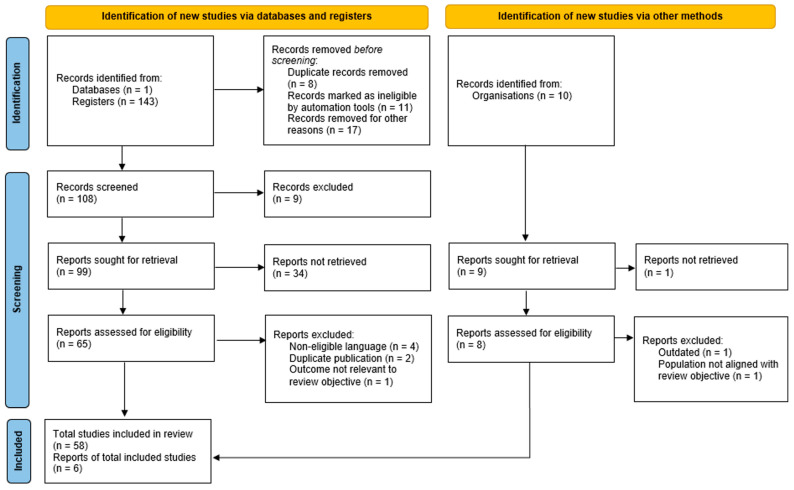
Flow diagram of the literature search and selection process for this narrative review. Adapted from the PRISMA 2020 flow diagram template [10] for illustrative purposes.

Given the heterogeneity of available evidence and the ethical constraints inherent to research involving pregnant patients with cancer, findings were synthesized narratively rather than through quantitative meta-analysis. The study selection process is summarized in an adapted flow diagram (Figure 1) to enhance transparency, although the present work does not constitute a formal systematic review with strict application of PRISMA criteria.

### 2.2. Eligibility Criteria

We included clinical practice guidelines, systematic reviews, observational cohort studies, registry-based studies, case series, and selected case reports addressing the diagnosis, treatment, and outcomes of cancer during pregnancy. Articles focusing on cancers diagnosed during gestation or within one year postpartum were considered eligible. Studies not published in English, Spanish, or Portuguese, editorials without original data, and reports lacking explicit relevance to pregnancy-associated cancer were excluded.

### 2.3. Data Synthesis

Given the heterogeneity of study designs and the ethical constraints inherent to this field, a quantitative meta-analysis was not pursued. Instead, findings were synthesized narratively and organized into thematic domains, including epidemiology, physiological adaptations of pregnancy, diagnostic challenges, systemic therapies, surgical and radiotherapeutic management, obstetric decision-making, placental and fetal metastasis, ethical considerations, and long-term maternal and offspring outcomes.

### 2.4. Ethical Considerations

This article is a narrative review based exclusively on previously published data. No new studies involving human participants or animals were conducted by the authors; therefore, approval from an ethics committee was not required.

### 2.5. Use of Artificial Intelligence Tools in Manuscript Preparation

During the preparation of this manuscript, the authors used ChatGPT (GPT-5.3, OpenAI; accessed in January 2026) to assist in the conceptual development and generation of Figure 3. The authors critically reviewed and edited the generated content and take full responsibility for the accuracy and integrity of the figure.

## 3. Epidemiology of Cancer in Pregnancy

Pregnancy-associated cancer, defined as cancer occurring during pregnancy or within one year after delivery, affects approximately 1 in 1000 pregnancies. Breast cancer is considered the most common type of cancer during pregnancy and represents the leading cause of cancer-related mortality among women during this stage of life. It primarily affects women aged 32 to 38 years and has an incidence of approximately 3 per 10,000 pregnancies, with a rising trend over recent years [11]. In addition, pregnancy-associated breast cancer accounts for 0.2–3.8% of all breast malignancies, with more than half of cases diagnosed in the postpartum period [12].

Cervical cancer represents a significant public health concern and has an estimated incidence during pregnancy ranging from 0.44 to 5.08 cases per 100,000 pregnancies. Although it is a preventable malignancy through human papillomavirus (HPV) vaccination and early screening programs, higher rates of cervical cancer during pregnancy are observed in regions with less organized primary healthcare systems, commonly found in countries with lower socioeconomic status [13].

The diagnosis of hematologic malignancies during pregnancy is rare. Among these, Hodgkin lymphoma (HL) accounts for approximately 6% of pregnancy-associated cancers, followed by non-Hodgkin lymphoma (NHL) at 5%, while leukemia and multiple myeloma are less frequent, representing approximately 4% and 0.1% of cases, respectively [14,15]. Malignant melanoma accounts for approximately 25% to 31% of cancer cases in pregnant women. Its incidence has increased over time, mainly due to the postponement of pregnancy to more advanced maternal ages [16,17].

Thyroid cancer during pregnancy has an estimated incidence of approximately 14 cases per 100,000 pregnant women and represents about 10% of all thyroid cancer cases in women of reproductive age. Among these, more than 70% are diagnosed after delivery [18,19].

Based on these epidemiological data, it is evident that although cancer during pregnancy is relatively rare, it constitutes a clinically relevant condition, particularly in light of the increasing incidence of malignancies among women of reproductive age associated with delayed motherhood.

## 4. Physiological Changes in Pregnancy and Implications for Cancer

Hormones produced by the corpus luteum during the early weeks of pregnancy play a fundamental role in maintaining gestation until placental takeover occurs. Estrogen contributes to trophoblastic invasion, placental development, and angiogenesis, whereas progesterone is essential for blastocyst implantation. Even before placental development, the trophoblast secretes hormones such as human chorionic gonadotropin (hCG), in addition to estrogen and progesterone. hCG plays a key role in implantation and nidation and suppresses myometrial contractions, thereby ensuring pregnancy maintenance [8].

This hormonal milieu may be associated with oncogenesis during pregnancy. Gestational hyperestrogenism, which is crucial for uterine growth, breast development, and systemic maternal adaptation, is also linked to several types of cancer. At the cellular level, estrogen stimulates cell proliferation and progression by promoting c-myc expression and cyclin D1 activation, as well as modulating apoptosis-related proteins such as Bcl-2 and Bcl-x, thereby creating favorable conditions for tumor growth and progression. Progesterone is also involved in mechanisms that promote the initiation and progression of tumor cells, particularly in hormone-dependent tissues, and hCG shares evolutionary similarities with carcinogenic pathways [20].

Among maternal physiological adaptations, hemodynamic changes may also impact cancer treatment. Structural changes in the left ventricle, activation of the renin–angiotensin–aldosterone system, and hormonal fluctuations result in increased plasma volume, increased cardiac output, and decreased systemic vascular resistance [21]. These hemodynamic changes during pregnancy, particularly plasma volume expansion, may affect pharmacokinetics and cancer treatment, as they occur alongside increased glomerular filtration and reduced plasma protein concentrations, directly influencing the pharmacokinetics and effectiveness of systemic therapies [8].

Furthermore, immunological modulation during pregnancy and carcinogenic pathways share similar strategies of immune tolerance and suppression. During pregnancy, the maternal immune system adapts to tolerate the semi-allogeneic fetus while maintaining defense against infectious agents. These adaptations include increased regulatory T lymphocytes, B lymphocytes, and a shift in immune response from Th1 to Th2 profiles [22,23]. Similarly, neoplastic cells promote the recruitment and expansion of B and T lymphocytes, increase the expression of PD-1 and PD-L1, and suppress immune responses, thereby favoring tumor survival, growth, and progression [24].

Finally, cancer-related symptoms during pregnancy often resemble physiological changes in gestation, such as fatigue, nausea, vomiting, abdominal pain, vaginal bleeding, and mastalgia. For example, fatigue, dyspnea, and night sweats may be interpreted as common pregnancy symptoms but are also characteristic of Hodgkin lymphoma. Likewise, breast nodules may be attributed to gestational mastopathy but are also a hallmark of breast cancer. Due to this symptom overlap, diagnosis and appropriate treatment are frequently delayed, adversely affecting disease prognosis [25,26] (Table 1).

## 5. Diagnostic Challenges

The diagnosis of cancer during pregnancy is frequently delayed due to the overlap between cancer-related symptoms and the physiological changes in gestation. Fatigue, nausea, vomiting, breast changes, and vaginal bleeding are symptoms that may occur concomitantly in both cancer and pregnancy. Reluctance to pursue adequate diagnostic investigation, particularly in younger women, who are often perceived as being at low risk for malignancy, further delays diagnosis and contributes to disease identification at more advanced stages [4,27]. In the case of breast cancer, for example, pregnant women have a 2.5-fold higher risk of being diagnosed at a more advanced stage compared with non-pregnant women [8].

Ultrasonography is considered the primary diagnostic modality for cancer during pregnancy, as it does not expose the patient to ionizing radiation. It is particularly important in the evaluation of breast and ovarian cancer. Moreover, it is regarded as a highly sensitive imaging technique, although its accuracy may be limited by the patient’s body habitus [4,28].

As a complementary modality, magnetic resonance imaging (MRI) is considered a highly effective and safe alternative for cancer diagnosis and staging, particularly when ultrasonography is inconclusive. MRI does not involve ionizing radiation, and there is no consistent evidence of teratogenic effects associated with the magnetic fields and radiofrequency waves used in clinical examinations. When clinically indicated, non-contrast MRI performed during pregnancy has not been associated with adverse fetal or neonatal outcomes, such as alterations in birth weight or impairments in neurological, motor, or auditory development [4].

The use of gadolinium-based contrast agents to enhance MRI, however, warrants additional caution. Gadolinium crosses the placenta, enters the fetal circulation, and may remain for prolonged periods in the amniotic fluid, subsequently being swallowed by the fetus. This raises theoretical concerns regarding toxicity and tissue deposition. Observational studies suggest a possible association between in utero exposure to gadolinium and an increased risk of rare adverse outcomes, including rheumatologic or inflammatory conditions and fetal death, although the available data are limited and subject to bias. Consequently, major scientific societies recommend that gadolinium contrast agents be avoided during pregnancy and reserved only for exceptional circumstances in which the anticipated diagnostic benefit clearly outweighs the potential risks and when the information obtained is expected to directly influence maternal or fetal clinical management [29,30].

With regard to computed tomography (CT), its use during pregnancy should be avoided, as it involves exposure to ionizing radiation. The total cumulative dose of ionizing radiation during pregnancy should remain below 100 mGy to reduce the risk of congenital malformations, intrauterine growth restriction, and fetal death. The risks associated with fetal exposure during imaging vary according to gestational age, radiation dose, and duration of exposure, with the first trimester representing a potentially higher-risk period [4,27].

In contrast, ^18F-FDG PET/CT (Positron Emission Tomography/Computed Tomography with Fluorine-18–labeled fluorodeoxyglucose) entails higher fetal exposure to radiation and radiopharmaceuticals, posing fetal risk throughout all trimesters. Fetal uptake of ^18F-FDG tends to concentrate mainly in the myocardium, brain, and urinary system and varies according to gestational age. Strategies to reduce fetal exposure include substitution with PET/MRI, adequate maternal hydration, increased urinary frequency, and bladder catheterization. Nevertheless, its use during pregnancy remains controversial and should be reserved only for exceptional circumstances [31].

The use of tumor markers during pregnancy presents considerable limitations, as these markers exhibit reduced sensitivity and specificity and may be physiologically elevated during gestation, complicating clinical interpretation. Tumor markers commonly used in pregnancy-associated malignancies, such as CA-125, alpha-fetoprotein (AFP), and β-hCG, may be elevated in normal pregnancies. CA-125, for example, may be increased during the first trimester and in the immediate postpartum period, while AFP is physiologically produced by the fetal liver, rendering its isolated use as a tumor marker during pregnancy unreliable [4,32].

**Figure 2:**
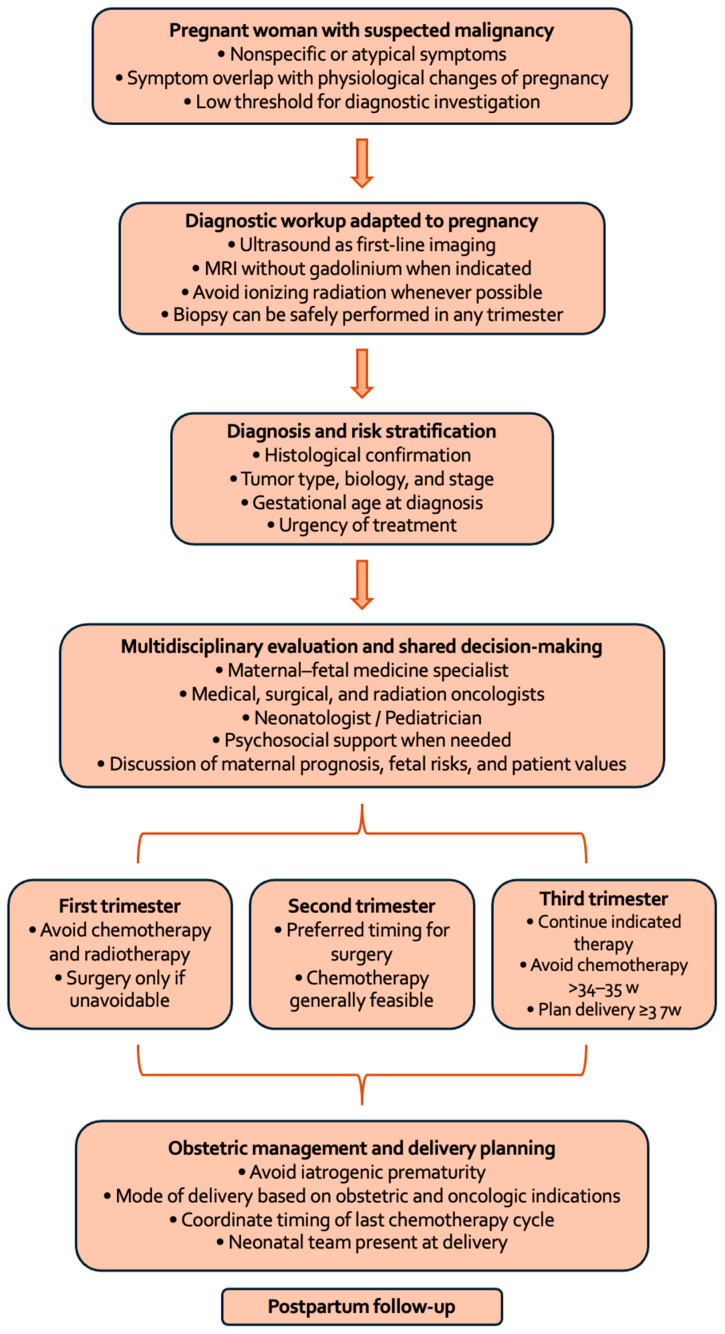
Multidisciplinary decision-making pathway for cancer diagnosed during pregnancy. Abbreviation: w, weeks of gestation.

Finally, when required, diagnostic confirmation of cancer during pregnancy can be achieved through biopsy procedures in any trimester and should not be postponed, as timely histopathological diagnosis is essential for appropriate staging and therapeutic planning. Core needle biopsy, for instance, is considered safe during pregnancy, and delaying diagnosis may result in disease identification at more advanced stages [4]. Figure 2 shows the multidisciplinary decision-making pathway for cancer diagnosed during pregnancy.

## 6. Principles of Oncologic Treatment During Pregnancy

The selection of an oncologic treatment plan in pregnant women must be individualized, considering cancer type, tumor biology, and disease stage. This process is further complicated by the influence of gestational age and the limited availability of robust data regarding the safety of most antineoplastic therapies during pregnancy. Therefore, when cancer is diagnosed during gestation, careful assessment of gestational age at diagnosis, as well as the clinical and biological characteristics of the tumor, is essential to guide therapeutic decision-making in a manner that is safe for both the mother and the fetus [2,33,34].

In general terms, oncologic treatment during pregnancy is preferable to both pregnancy termination and elective preterm delivery. Pregnancy termination has not been shown to confer maternal prognostic benefit and should not be indicated solely for this purpose, given the paucity of studies and the lack of evidence demonstrating improved maternal outcomes to date [8,9,33]. Similarly, elective preterm delivery should be avoided whenever possible, since complications related to prematurity represent the leading cause of adverse neonatal outcomes in this population, regardless of chemotherapy exposure [34].

Whenever feasible, cancer treatment during pregnancy should follow the same protocols used in non-pregnant women of similar age, as this approach has been associated with comparable prognoses between pregnant and non-pregnant patients. In this context, management should prioritize the minimization of iatrogenic prematurity, reinforcing the growing recognition that effective oncologic treatment is compatible with the continuation of pregnancy [3,8].

According to the literature, chemotherapy and targeted therapies should be avoided during the first trimester; however, many oncologic therapies can be safely administered during the second and third trimesters.

Surgical intervention during pregnancy is feasible and, when indicated, should be performed under the supervision of surgical and anesthetic teams experienced in the physiological changes in pregnancy. In general, surgical treatment is not contraindicated at any gestational age. However, in the management of gynecologic malignancies, surgery is preferably performed early in the second trimester, a period associated with a lower risk of spontaneous abortion and improved technical conditions due to uterine size [33].

Radiotherapy should generally be avoided throughout pregnancy, except in rare cases in which the potential benefits clearly outweigh the risks [34]. Breastfeeding provides essential benefits for both mother and newborn; however, its safety following cancer treatment during pregnancy depends on the type and timing of therapy [2].

Cancer treatment during pregnancy represents an extremely delicate situation that requires meticulous, specialized, and individualized care [34]. A multidisciplinary team is strongly recommended to assess each case and to guide the patient and her family throughout the processes of counseling, diagnosis, and treatment [2]. The multidisciplinary team should include, at a minimum, a maternal–fetal medicine specialist, clinical oncologist, perinatologist, gynecologic oncologist, pediatrician, radiation oncologist, and psychologist or social worker. Other professionals, such as a geneticist or clinical pharmacologist, may provide valuable contributions, and depending on the type of malignancy, the team may be expanded to include surgeons, hematologists, or other specialists as needed [9]. Management in tertiary care centers with access to specialized resources capable of addressing all stages of treatment is recommended to ensure comprehensive, high-quality care for both the pregnant woman and the fetus. Furthermore, follow-up should respect maternal autonomy and prioritize shared decision-making, supported by clear and continuous communication between the healthcare team and the patient, to optimizing maternal and fetal outcomes [33] (Table 2).

### 6.1. Chemotherapy in Pregnancy

The safety of chemotherapy during pregnancy is strongly related to the timing of fetal exposure. In pregnant women with cancer who require systemic therapy and choose to continue the pregnancy, conventional cytotoxic chemotherapy is recommended to be avoided during the first trimester due to the high risk of teratogenic and abortifacient effects [4]. Although pregnant women are systematically excluded from most clinical trials and available evidence regarding the teratogenic risks of chemotherapy is primarily derived from case reports, animal models, and small-scale observational studies [34], the literature consistently demonstrates a strong association between first-trimester chemotherapy exposure and the occurrence of congenital malformations, particularly when exposure takes place during fetal organogenesis. Therefore, whenever clinically feasible, the initiation of chemotherapy should be postponed until after the 12th–14th week of gestation, especially in early-stage cancers, to allow completion of organogenesis and reduce adverse fetal outcomes [2,33].

After the 12th–14th week of gestation, the risk of fetal malformations decreases substantially. Studies indicate that from this period onward, the incidence of congenital malformations in fetuses exposed to chemotherapy is similar to that observed in the general population without cancer and without exposure to chemotherapeutic agents. Although modest associations with prematurity, intrauterine growth restriction, and low birth weight have been reported, these findings are limited by the lack of appropriate control groups [33,34].

Given the potential maternal benefit of timely oncologic treatment, exposure to chemotherapy during the second and third trimesters is considered justifiable in many cases. Accordingly, chemotherapy may be administered starting in the second trimester [9]. After the 34th–35th week of gestation, clinical consensus tends to contraindicate the continuation of chemotherapy. It is recommended that the last dose be scheduled two to four weeks prior to delivery [4] to allow adequate recovery of maternal and fetal bone marrow following the final cycle, thereby reducing the risk of maternal and neonatal myelosuppression during the peripartum period and promoting improved maternal–fetal outcomes. In the postpartum period, breastfeeding should be contraindicated during chemotherapy due to the risk of neonatal exposure to cytotoxic agents [2,8,33].

Physiological changes in pregnancy, particularly those more pronounced in the third trimester, may significantly affect pharmacokinetic parameters. Increased renal clearance and hepatic metabolic activity, alterations in gastrointestinal absorption, and expansion of plasma volume—characteristic features of pregnancy—may influence drug distribution volume, absorption, and, consequently, fetal exposure to cytotoxic agents, potentially compromising both the efficacy and safety of systemic oncologic treatment [9,33,34]. Despite the absence of robust studies defining pregnancy-specific chemotherapy pharmacokinetics, current recommendations advise dosing based on actual body weight, without dose adjustments solely due to pregnancy [4,8].

In general, chemotherapy protocols used during pregnancy should follow the same guidelines applied to non-pregnant patients [2].

#### 6.1.1. Platinum-Based Agents

Platinum-based agents, particularly cisplatin and carboplatin, are considered relatively safe when administered during the second and third trimesters, although they are associated with an increased risk of preterm delivery and low birth weight [1,4,35]. Ototoxicity is a recognized adverse effect of these agents and is more frequently associated with cisplatin than with carboplatin. Reports of neonatal hearing loss following in utero exposure highlight the need for systematic auditory follow-up of exposed newborns [4,36]. Regarding oxaliplatin, although safety data remain limited compared with cisplatin and carboplatin, case reports describing its use in the treatment of colorectal cancer during pregnancy have reported favorable neonatal outcomes without evidence of congenital anomalies [4,35]. Vinca alkaloids, including vinblastine and vincristine, as well as vinorelbine in selected cases, are also considered safe during the second and third trimesters [35].

#### 6.1.2. Taxanes

With respect to taxanes, particularly paclitaxel and docetaxel, the literature indicates low placental transfer and low toxicity, with no significant increase in congenital anomalies or developmental delays, supporting their safety when used during the second and third trimesters of pregnancy [1,4,5].

#### 6.1.3. Anthracyclines

Anthracyclines, such as doxorubicin and epirubicin, are among the most frequently used chemotherapeutic agents during pregnancy and play an important role in the treatment of hematologic malignancies and breast cancer. In contrast, idarubicin raises greater concern due to its teratogenic potential, even when administered after the first trimester. Its high lipophilicity promotes increased fetal accumulation and exposure, which has been associated with a higher incidence of congenital anomalies and fetal death; therefore, its use should be avoided [2,4,35].

#### 6.1.4. Alkylating Agents

Alkylating agents, including cyclophosphamide, ifosfamide, and dacarbazine, have been used with relative safety in standard treatment regimens during pregnancy, with cyclophosphamide being the agent supported by the most consistent safety data. Although occasional reports of preterm birth and low birth weight exist, long-term developmental outcomes are generally favorable. In contrast, the use of high-dose alkylating agents, such as busulfan, ifosfamide, and melphalan, as well as intensive combination regimens such as BEACOPP, remains contraindicated or strongly discouraged at any stage of pregnancy due to documented embryotoxicity [2,4]. Methotrexate is contraindicated throughout pregnancy, particularly during the first trimester, because of its well-established teratogenic effects, including a high risk of spontaneous abortion, fetal death, and congenital anomalies [4].

#### 6.1.5. Combination Chemotherapy Regimens

Regimens such as CHOP (cyclophosphamide, doxorubicin, vincristine, and prednisone), ABVD (doxorubicin, bleomycin, vinblastine, and dexamethasone), and FAC/FEC/AC/EC (5-fluorouracil, doxorubicin, epirubicin, and cyclophosphamide) are commonly used during pregnancy. In the CHOP regimen, prophylaxis for tumor lysis syndrome should be considered. The ABVD regimen requires monitoring of fetal growth and maternal pulmonary function due to bleomycin exposure. The FAC/FEC/AC/EC regimens require strict maternal surveillance because of the high risk of neutropenia [2].

#### 6.1.6. Supportive Medications

In cases of myelosuppressive chemotherapy, the use of granulocyte colony-stimulating factor (G-CSF) may be considered to reduce the risk of febrile neutropenia, following individualized assessment of risk factors, gestational age, and the risk–benefit balance [4]. Supportive medications also play a fundamental role during systemic oncologic treatment. For the management of nausea, a commonly reported symptom, ondansetron has demonstrated greater efficacy compared with metoclopramide and the combination of doxylamine and pyridoxine, without consistent evidence of an increased incidence of congenital malformations when used during pregnancy [35].

Although most population-based cohorts and meta-analyses do not demonstrate a meaningful increase in overall congenital malformations associated with ondansetron use during pregnancy [37,38], large observational studies and regulatory reassessments have identified a small but consistent risk signal in some analyses, particularly for oral clefts—especially when exposure occurs during the first trimester—and, to a lesser extent, for specific cardiac defects, such as ventricular septal defects.

In the largest available population-based study, using Medicaid data, first-trimester exposure to ondansetron was not associated with an increased risk of overall or cardiac malformations after adjustment for relevant confounders; however, a small absolute increase in the risk of oral clefts was observed. Other studies based on administrative (“claims”) databases and analyses focused on specific outcomes have reported heterogeneous associations with cardiac and orofacial defects, generally of modest magnitude and without consistency across different methodological designs [39,40,41,42]. This body of evidence has led European regulatory authorities to recommend caution and, whenever possible, avoidance of ondansetron use during the first trimester of pregnancy, acknowledging a potential low absolute increase in the risk of oral clefts. In the context of pregnancy-associated cancer, however, this issue must be interpreted with particular care. Pregnant women undergoing oncologic treatment frequently experience severe nausea and vomiting related not only to pregnancy itself, but also to cytotoxic chemotherapy, supportive therapies, and the metabolic and emotional burden of the disease. In such scenarios, inadequate symptom control may result in dehydration, electrolyte disturbances, weight loss, deterioration of nutritional status, and the need for interruption or delay of oncologic treatment, potentially negatively affecting maternal prognosis.

Moreover, the severity of hyperemesis and chemotherapy-induced nausea constitutes an important indication bias in observational studies, as more severe clinical presentations are more likely to require ondansetron and are themselves associated with poorer pregnancy outcomes. Therefore, in the oncologic management of pregnant patients, ondansetron should be regarded as an effective therapeutic option, generally reserved for situations of failure or intolerance to first-line therapies, such as metoclopramide and the combination of doxylamine and pyridoxine. Its use—particularly after the first trimester—may be clinically justifiable when based on an individualized risk–benefit assessment, at the lowest effective dose, and within a shared decision-making framework. Thus, rather than an absolute contraindication, the use of ondansetron during pregnancy in oncologic patients should be interpreted in light of the low absolute risk described, the methodological limitations of the available evidence, and the need to ensure adequate clinical support for the safe continuation of maternal treatment.

The nature and mechanisms by which chemotherapy induces congenital malformations are not fully understood and involve multiple factors, including genetic susceptibility, timing of chemotherapy exposure, and the specific agents used [33,43]. To date, there is no robust evidence that in utero exposure to chemotherapy definitively results in impaired postnatal growth or long-term cognitive or cardiac dysfunction [34,36]. Nevertheless, it is recommended that newborns exposed to intrauterine oncologic therapies be monitored carefully in order to identify potential treatment-related complications and ensure early detection and intervention [4].

A prospective cohort study by Amant et al. [44], involving 129 children born to mothers with cancer during pregnancy (74% exposed to intrauterine chemotherapy), found no significant differences in early childhood health outcomes compared with controls. Additional evidence indicates that prematurity—rather than intrauterine exposure to chemotherapy—constitutes an independent factor associated with poorer cognitive outcomes [45]. Consistently, a post hoc analysis by Passera et al. demonstrated that differences in verbal intelligence were more pronounced among children who had lost their mother to cancer, rather than among those exposed to intrauterine chemotherapy [46]. Nevertheless, despite generally favorable long-term prognoses, the available data remain limited, and further studies are required for a more comprehensive assessment of the long-term safety of these treatments [36].

### 6.2. Surgery During Pregnancy

Surgery constitutes a fundamental component of oncologic treatment and, when performed by experienced multidisciplinary teams, can be safely undertaken during all trimesters of pregnancy. The choice of surgical techniques and associated outcomes are directly related to gestational age and the stage of fetal development. When delivery is imminent, postponement of the procedure to the postpartum period may be considered; however, when surgery is unavoidable, specific strategies should be adopted to optimize maternal and fetal outcomes [4,47]. It is generally considered appropriate to avoid surgical procedures during the first trimester, as this period is associated with a higher risk of spontaneous abortion and fetal complications, corresponding to the phase of organogenesis. During this stage, the embryo is particularly vulnerable to external insults, including surgical stress, anesthetic effects, and potential maternal surgical complications such as hypotension, hypoxemia, and infection, which may compromise fetal development [48,49].

Overall, the early second trimester is regarded as the most appropriate period for performing surgery during pregnancy, particularly intra-abdominal procedures, as the uterus remains relatively small and the risk of spontaneous abortion is reduced. For surgeries performed after 20 weeks of gestation, left lateral tilt positioning is recommended to avoid compression of the inferior vena cava and to maintain adequate cardiac preload [2,4,47]. Surgical approaches vary significantly according to cancer type, stage, location, and gestational age.

In the context of breast cancer, modified radical mastectomy and breast-conserving surgery, including sentinel lymph node biopsy (SLNB) using technetium, can be safely performed throughout pregnancy. However, the use of blue dye in SLNB remains controversial due to limited data regarding fetal safety [4,34]. In contrast, certain components of breast cancer management, such as breast reconstruction, should be postponed until the postpartum period [2].

For cervical cancer, the literature indicates that conization alone is considered safe and sufficient during pregnancy in cases of stage IA or IA1 cervical cancer diagnosed before 25 weeks of gestation, despite an increased risk of bleeding that intensifies as pregnancy progresses. When indicated, simple trachelectomy is also considered a safe option and is preferable to radical trachelectomy, which is associated with high rates of obstetric and surgical complications. Lymphadenectomy represents another feasible option, depending on disease stage, when performed up to approximately 24 weeks of gestation. At more advanced gestational ages or in more advanced stages of disease, neoadjuvant chemotherapy constitutes the only therapeutic strategy capable of preserving pregnancy [2,8].

In early-stage ovarian cancer, conservative surgery during pregnancy is frequently described, with definitive staging deferred to the postpartum period. In cases of advanced disease with peritoneal dissemination, where pregnancy preservation generally precludes standard treatment and is associated with worse maternal prognosis, available evidence supports strategies such as pregnancy termination or, when continuation of pregnancy is desired, the administration of neoadjuvant chemotherapy until fetal maturity, followed by surgery in the late postpartum period [2,4]. Surgical intervention without delay, preferably before 20 weeks of gestation, is recommended to reduce the risk of disease progression [34].

Regarding thyroid cancer, surgical intervention performed preferentially during the second trimester minimizes the risks of maternal hypothyroidism and fetal developmental impairment and is not associated with adverse neonatal outcomes. In melanoma, surgical excision represents the treatment of choice and can be safely performed during pregnancy. Wide local excision, when indicated, may require fetal monitoring. Sentinel lymph node biopsy is prognostically relevant and, when indicated, should be performed using technetium [4,34].

From an anesthetic perspective, careful management of anesthesia duration, rigorous monitoring of maternal condition, and effective postoperative pain control are essential. Hemodynamic and physiological changes inherent to pregnancy significantly affect perioperative monitoring, making vigilant surveillance of hemodynamic parameters indispensable for preventing hypoxia, hypotension, and hypoglycemia, thereby ensuring maternal and fetal well-being [47]. In the postoperative period, inadequately controlled pain may induce uterine contractions and increase the risk of preterm labor. However, analgesic management in this context remains clinically challenging due to the limited availability of options proven to be safe during pregnancy. Commonly used analgesics, such as dipyrone and paracetamol, are considered safe during pregnancy, as is the short-term use of opioids, which is generally well tolerated by the fetus. In contrast, the use of nonsteroidal anti-inflammatory drugs (NSAIDs) requires caution [4,47]. Evidence suggests possible embryotoxicity when NSAIDs are used during the first trimester; an increased risk of fetal renal dysfunction and oligohydramnios after 20 weeks; and, from 30 weeks onward, an additional risk of premature closure of the ductus arteriosus. Therefore, although NSAID use between 12 and 20 weeks is not formally contraindicated, restriction to refractory cases and situations of extreme necessity is recommended [50].

Whenever possible, locoregional anesthesia should be prioritized over general anesthesia due to its safety and efficacy benefits. When general anesthesia is required, rapid-sequence induction is recommended [2,51]. Despite relatively favorable safety profiles, surgical interventions during pregnancy remain associated with risks, including preterm birth, fetal distress, and spontaneous abortion. In addition to the risk of fetal loss, surgery may lead to hypercapnia, perforation of the enlarged uterus, and reduced uterine blood flow due to increased intra-abdominal pressure and the use of carbon dioxide [2,47]. When surgical intervention is necessary, the healthcare team should anticipate potential adverse outcomes and consider strategies to reduce fetal morbidity once viability is achieved. Preoperative administration of corticosteroids to promote fetal lung maturation and the use of tocolytics for up to 48 h postoperatively in cases involving intraoperative uterine manipulation may be considered to reduce the risk of preterm labor and its consequences [2,4]. Furthermore, evidence indicates that maintaining intraperitoneal pressure at or below 15 mmHg and limiting operative time to less than 90 min are associated with reduced maternal and fetal complications [4].

For surgical procedures performed after fetal viability, intraoperative fetal heart rate monitoring should be discussed in advance with the patient and conducted in coordination among surgical, obstetric, and neonatal teams to define appropriate management in the event of nonreassuring patterns during surgery. In addition, postoperative fetal assessment using Doppler velocimetry is recommended [5]. Maternal clinical stability remains the primary determinant of fetal well-being. Thus, awareness of the risks associated with surgery during pregnancy, combined with rigorous maternal monitoring, is fundamental to ensuring the safety of the maternal–fetal dyad [47].

Pregnancy itself should also be considered a prothrombotic state, and when combined with other high thrombotic risk factors such as surgery, immobility, and malignancy, the risk of thromboembolic events increases significantly, making the implementation of venous thromboembolism (VTE) prevention strategies essential. Low-molecular-weight heparin plays a well-established role in reducing the incidence of thromboembolic complications; therefore, prophylactic administration is recommended. Additional strategies, such as the intraoperative use of graduated compression stockings and intermittent pneumatic compression devices, may provide valuable support and should be considered [2,47,51].

Studies demonstrate that both laparoscopy and laparotomy can be performed during pregnancy, with the choice of approach determined by factors such as gestational age, procedural complexity and expected duration, and surgeon experience [4]. Compared with laparotomy, laparoscopy is associated with reduced blood loss, shorter hospital stay, and a lower incidence of wound-related complications. When performed up to the second trimester, laparoscopy represents a feasible and safe option, allowing the benefits of minimally invasive surgery without compromising maternal or fetal safety [2,52].

### 6.3. Radiotherapy

Human embryonic and fetal development is highly sensitive to ionizing radiation, rendering radiotherapy during pregnancy a complex and widely debated issue. Available evidence regarding its safety and maternal–fetal outcomes is predominantly derived from retrospective studies, case reports, and expert opinion, with a paucity of high-quality prospective data. In general, radiotherapy should be avoided during pregnancy. However, when clinically justified, particularly in situations where treatment delay would pose a significant risk to the patient, it may be performed with appropriate protective measures. Despite technological advances and available shielding strategies, the potential for short- and long-term adverse fetal effects necessitates restricting its indication to carefully selected patients following thorough multidisciplinary evaluation and rigorous consideration of safer therapeutic alternatives whenever available [2].

In general, it is recommended that the cumulative fetal radiation dose should not exceed 100 mGy, as exposures above this threshold have been associated with dose-dependent neurodevelopmental effects, including reductions in intelligence quotient and severe intellectual disability [2,4,50]. Fetal radiation dose primarily results from internal scattered radiation, which is influenced by the total administered dose, number of monitor units, irradiation field size, distance between the uterus and beam edges, equipment characteristics, and radiation transmitted through multileaf collimators, filters, blocks, or other accessories [52]. Nevertheless, fetal risk is not determined solely by radiation dose, but is also influenced by gestational age and the anatomical region exposed to irradiation [53].

Embryonic and fetal sensitivity to ionizing radiation varies according to gestational age, making this factor essential in risk assessment during pregnancy. During the preimplantation period, the embryo exhibits high radiosensitivity, with implantation failure or early embryonic death representing the primary consequences of exposure; however, this risk is considered extremely low at doses below 100 mGy [2,53]. The period of organogenesis (weeks 2–7) and early brain development (weeks 8–15) represents the most critical phase with respect to radiation exposure. During this stage, there is an increased risk of spontaneous abortion, fetal death, and congenital malformations, particularly of neurological origin. The most frequently described effects include microcephaly and cognitive impairment, not necessarily interrelated, as well as intrauterine growth restriction. Evidence indicates a marked increase in these risks when embryonic or fetal doses exceed 100 mGy, with exposures above 300–500 mGy associated with a high incidence of intellectual disability, microcephaly, congenital malformations, and early embryonic death. Considering these findings, radiotherapy during this period is rarely indicated, although it may be considered in exceptional circumstances with rigorous dosimetric planning and careful patient selection [2,4].

During the second trimester, fetuses become relatively more resistant to the teratogenic effects of radiation, although susceptibility to neurodevelopmental alterations persists. In this period, doses between 100 and 150 mGy are associated with a low risk of malformations, whereas exposures exceeding this threshold increase the risk of microcephaly and cognitive impairment. In addition, the increasing proximity of the fetus to irradiation fields as gestation advances may increase exposure, with reported risks including growth restriction, cataracts, sterility, radiation hypersensitivity syndrome, and radiation-induced cancer. Nonetheless, radiotherapy may be feasible during the second trimester when carefully planned. In the third trimester, fetuses exhibit lower vulnerability to teratogenic radiation effects, and exposure is less frequently associated with adverse developmental outcomes. Studies suggest that doses below 500 mGy during this period are not associated with a significant increase in cognitive impairment, although isolated cases of growth abnormalities, microcephaly, and radiotherapy-induced preterm delivery have been reported. Additionally, the estimated risk of radiation-induced childhood cancer is substantially lower when exposure occurs during the third trimester, making this period relatively safer for potential fetal exposure [2,4].

The risks of radiotherapy during pregnancy vary significantly according to tumor location and the proximity of the irradiation field to the uterus and fetus. Studies involving irradiation of supradiaphragmatic malignancies, such as breast cancer, head and neck tumors, and Hodgkin lymphoma, demonstrate that fetal dose may remain within acceptable limits when rigorous planning and protective measures are employed, particularly when the uterus is distant from the treatment field [53]. In such scenarios, radiotherapy may be considered feasible, provided that fetal exposure is maintained below 100 mGy and confirmed through phantom-based dosimetric estimates, ideally complemented by in vivo measurements [8,53]. In contrast, subdiaphragmatic irradiation, particularly pelvic radiotherapy, is considered high risk during pregnancy, as it typically results in significant fetal exposure. For this reason, pelvic radiotherapy is not recommended during pregnancy, and continuation of pregnancy often needs to be discussed in cases of locally advanced malignancies requiring immediate radiotherapeutic treatment [36,53]. Whenever possible, postponement of radiotherapy until the postpartum period is recommended, especially for pelvic tumors [2].

When upper-body radiotherapy is required, implementation of shielding strategies is essential. These may include more complex protective structures than conventional lead aprons, such as tertiary shielding bridges or walls. However, the effectiveness of these strategies may be limited by continuous changes in uterine position and size throughout gestation, necessitating frequent adjustments and close collaboration between radiation oncologists and medical physicists [2,53]. Alternative techniques, such as intraoperative radiotherapy and the use of proton or electron beams, may significantly reduce fetal dose in selected situations. Proton therapy, in particular, offers potential advantages over three-dimensional conformal radiotherapy and volumetric modulated arc therapy due to its sharp dose gradient and minimal exit dose, although logistical limitations and dosimetric uncertainties remain, particularly regarding the effectiveness of fetal shielding [4]. Pre-treatment estimation of fetal dose using phantom models is recommended, with confirmation through in vivo dosimetry during treatment, as well as the possibility of adjustments to the radiotherapy plan—such as modification of field size, beam angles, and radiation energy—whenever feasible [36,53].

### 6.4. Targeted Therapy, Immunotherapy, and Novel Agents

Recent advances in oncologic treatment, with the incorporation of targeted therapies, immunotherapies, and novel agents, have significantly transformed the prognosis of several malignancies in women of reproductive age. However, the application of these strategies during pregnancy remains particularly challenging due to their specific mechanisms of action, the potential for interference with critical embryofetal developmental pathways, and the scarcity of robust clinical data on maternal–fetal safety. Unlike conventional cytotoxic chemotherapy, whose risk profile is now relatively well characterized from the second trimester onward, targeted and immunomodulatory therapies act on molecular signaling pathways, growth factor receptors, and immune mechanisms that also play central roles in placentation, angiogenesis, and maternal–fetal immune tolerance. Consequently, their use during pregnancy requires extreme caution and is generally discouraged in the absence of clearly outweighing maternal benefits relative to potential fetal risks.

Anti-HER2 therapy, such as trastuzumab, is contraindicated during pregnancy due to the risk of oligohydramnios, anhydramnios, congenital respiratory disorders, neonatal renal failure, pulmonary hypoplasia, and neonatal death, particularly when exposure occurs during the second and third trimesters. Other anti-HER2 agents are likewise discouraged owing to the lack of evidence demonstrating safety during pregnancy [27,54].

Tyrosine kinase inhibitors, such as imatinib, block enzymes involved in tumor proliferation and survival. Imatinib directly inhibits tyrosine kinase activity by binding to BCR-ABL, c-KIT, and platelet-derived growth factor (PDGF), thereby blocking the transmission of proliferative signals to the nucleus and inducing cellular apoptosis [34]. These agents are widely used in chronic myeloid leukemia and are also contraindicated during pregnancy, particularly in the first trimester. Reported adverse outcomes include fetal hydrops, polyhydramnios, and threatened preterm labor [55].

Immune checkpoint inhibitors (anti–PD-1, anti–PD-L1, and anti–CTLA-4) also represent a class of agents associated with significant risks during pregnancy and should therefore be avoided, particularly during the second and third trimesters, when transplacental antibody transfer is high. Their use has been associated with an increased risk of miscarriage, intrauterine growth restriction, prematurity, placental insufficiency, enterocolitis, and congenital hypothyroidism [4,56].

The main evidence gaps regarding the use of targeted therapies during pregnancy stem primarily from the lack of randomized clinical trials. Current evidence is largely based on case reports, case series, and retrospective studies, which provide limited information on true long-term fetal outcomes. Moreover, many studies derive their findings from preclinical animal models, further limiting the direct applicability of these results to human populations (Table 3). In addition, the absence of prospective studies and the limited duration of follow-up for exposed offspring further complicate the assessment of long-term safety. Consequently, current clinical guidance regarding these agents during pregnancy is largely based on precautionary principles rather than robust clinical evidence.

## 7. Obstetric Management

The timing of delivery should be coordinated by a multidisciplinary team comprising obstetricians, oncologists, surgical oncologists, and neonatologists. According to most current guidelines, the goal is to plan delivery from 37 weeks of gestation onward to reduce risks and complications associated with prematurity, while also avoiding adverse outcomes related to post-term pregnancy. Additionally, to minimize maternal and fetal hematologic toxicity, chemotherapy should be discontinued between 34 and 35 weeks of gestation, and delivery should be planned 1 to 3 weeks thereafter [4,28].

In women with cancer during pregnancy, cesarean delivery rates are substantially higher than those observed in the general obstetric population, frequently exceeding 50–60% in large cohorts and international registries. This trend primarily reflects iatrogenic decisions related to delivery planning, the need to synchronize obstetric management with oncologic treatment, and the higher incidence of elective prematurity. However, according to guidelines from the American Society of Clinical Oncology (ASCO) and the European Society for Medical Oncology (ESMO), vaginal delivery remains the preferred mode of birth whenever there are no obstetric or oncologic contraindications, as there is no evidence of maternal or fetal benefit associated with routine cesarean section in these cases. Cesarean delivery should therefore be reserved for standard obstetric indications or specific oncologic situations, particularly in cases of cervical, vaginal, and vulvar cancers, in which an abdominal approach is generally recommended [4,11,36].

Due to the increased risk of prematurity, antenatal corticosteroids remain the standard treatment to promote fetal lung maturation. A single course of corticosteroid therapy reduces the incidence of neonatal respiratory distress syndrome, intraventricular hemorrhage, and neonatal mortality.

The risk of intrauterine growth restriction is a frequent complication in pregnancies complicated by cancer. In such cases, it is recommended that from 22 to 24 weeks of gestation onward, patients undergo evaluation every 3–4 weeks to monitor fetal growth, amniotic fluid volume, and Doppler velocimetry [4,28].

After delivery, resumption of systemic therapy may occur 1–2 weeks postpartum, and breastfeeding should be assessed on an individual basis, considering the timing of the last administered dose and the need for early reinitiation of treatment [11]. The decision to breastfeed should respect the patient’s preferences, clinical and practical feasibility, and the risks of therapeutic agent transfer through breast milk. In addition, consideration should be given to the timing of delivery, the systemic agent used, and the drug’s half-life. In cases in which immediate resumption of treatment is required, patients may express and discard breast milk to preserve lactation and resume breastfeeding later. Concurrently, in patients undergoing breast radiotherapy, breastfeeding may be continued from the unaffected breast [11,28].

## 8. Placental and Fetal Metastasis

Placental metastasis is a rare but well-documented event across a wide range of malignant neoplasms. This dissemination occurs predominantly via the hematogenous route and is generally associated with the presence of systemic metastatic disease. Accordingly, the literature indicates that the presence of distant maternal metastases at the time of delivery constitutes an important risk factor for placental involvement. Although several tumors may involve the placenta, the most frequently reported are malignant melanoma (≈30%), followed by lung cancer (≈12%) and breast cancer (≈12%) [2,9,57].

The true frequency of placental involvement is difficult to determine, as histopathological examination of the placenta is not routinely performed in pregnancies complicated by cancer and lesions may be focal. Despite its rarity, placental involvement is clinically relevant, as it may represent the first sign of fetal metastasis, a condition associated with an extremely poor prognosis. Maternal prognosis in these cases is also unfavorable, with mortality rates of approximately 81%, largely attributable to the high frequency of concomitant distant metastases, observed in more than 90% of affected patients [2,57].

A particular consideration in this context is non-molar gestational choriocarcinoma, a rare but biologically aggressive entity that originates from prior gestational trophoblastic tissue and retains fetal genetic material, conferring unique characteristics to its pattern of dissemination. Unlike most maternal malignancies, gestational choriocarcinoma has a fetoplacental origin, with marked hematogenous tropism and high invasive potential, facilitating placental involvement and, in exceptional circumstances, transplacental transmission to the fetus. Although extremely rare, cases of fetal metastasis associated with gestational choriocarcinoma are well documented in the literature and typically present as aggressive metastatic disease in the neonatal period, frequently involving the liver, lungs, and brain.

In these scenarios, the placenta may act simultaneously as the primary site and as a route of tumor dissemination, rendering histopathological placental examination particularly crucial. Early identification of placental involvement may enable targeted neonatal surveillance and timely diagnosis of fetal disease, a condition associated with a guarded prognosis but potentially amenable to treatment when recognized early. Thus, non-molar gestational choriocarcinoma represents a biologically distinct exception within the spectrum of pregnancy-associated malignancies, reinforcing the importance of systematic placental evaluation and specialized neonatal follow-up whenever this diagnostic hypothesis is considered [58,59,60,61].

Placental dissemination from maternal cancer is influenced by the presence of a partial maternal–fetal barrier, the protective role of the fetal immune system, and the anatomical characteristics of the placenta. These factors help explain the more frequent localization of metastases within the maternal intervillous space and, to a lesser extent, within the placental villi. Intravillous involvement is rare but represents invasion of the fetal compartment of the placenta and is strongly associated with fetal involvement. Fetal metastases, although uncommon, are reported in approximately 22–50% of cases with villous invasion, a condition considered necessary for fetal dissemination. Identification of placental metastases may occur through macroscopic inspection; however, in many cases, detailed microscopic evaluation is required, as involvement may be focal and affect the intervillous space, villi, umbilical cord, or fetal vessels. Although the quality of evidence supporting routine histological evaluation of the placenta in pregnant women with cancer is generally low—due to the rarity of cases and reliance on case reports and series—the available data reinforce the importance of performing histopathological examination of the placenta in all cancers diagnosed during pregnancy [8,58].

In this context, placental histopathological evaluation is considered essential, particularly in patients with melanoma or metastatic disease, as it may identify previously unrecognized tumor dissemination, contribute to maternal restaging, and appropriately guide neonatal assessment and follow-up. When placental metastases are confirmed, comprehensive reassessment of maternal oncologic staging is recommended, along with referral of the newborn for specialized follow-up with a neonatologist and pediatric oncologist to exclude fetal metastasis. Despite its clinical relevance, most newborns with documented placental involvement do not exhibit evidence of disease at birth or during follow-up, with absence of neonatal involvement reported in approximately 79.5% of cases with known infant outcomes [4,58].

## 9. Ethical, Legal, and Psychosocial Considerations

Women diagnosed with cancer during pregnancy tend to experience substantial psychological distress, arising from the combination of a serious illness in young adulthood and the inherent challenges of pregnancy [7]. Concerns regarding fetal health, the oncologic diagnosis, therapeutic options, and pregnancy outcomes highlight the characteristic maternal dilemmas of this condition [2]. In this setting, empathetic engagement by healthcare professionals and effective communication are essential to validate patients’ concerns, reduce emotional distress, and promote informed, values-concordant decision-making. Shared decision-making plays a central role by ensuring patient autonomy through clear presentation of therapeutic options, including their benefits, risks, and uncertainties, allowing the pregnant woman to express her goals, such as continuation of pregnancy, fertility preservation, or prioritization of maternal health [4].

Ethical decision-making represents a central component in the management of cancer during pregnancy. Clinical decisions must balance maternal prognosis, fetal safety, and patient autonomy. Although some authors consider counseling regarding pregnancy termination to be feasible in specific situations, such as aggressive or advanced-stage cancers diagnosed early in pregnancy, the literature lacks high-quality evidence to support this approach. Accordingly, when therapeutic options compatible with pregnancy continuation exist or when treatment initiation can be postponed, pregnancy termination is not recommended solely to improve maternal prognosis. Decisions in this context are complex and frequently influenced by religious, cultural, and personal beliefs, as well as factors such as family dynamics, community influence, and trust in healthcare professionals; in some cases, pregnancy termination is declined even in the presence of significant maternal risk [8,33]. Comprehensive patient counseling is therefore essential. Pregnant patients should receive clear information regarding available therapeutic options, potential maternal benefits, fetal risks, and existing uncertainties. These dilemmas become particularly critical when urgent oncologic therapy is required but contraindicated due to pregnancy, especially when the fetus is not viable. In addition, legal restrictions on pregnancy termination impose further challenges on clinical practice, directly affecting medical counseling and the actions of oncologists in settings where termination for medical indications may be limited or unavailable [33].

Planning for post-treatment follow-up should begin during the active phase of oncologic care, with the aim of ensuring a smooth transition to long-term surveillance, including postpartum maternal health monitoring, assessment of child development, and discussion of fertility preservation strategies [4]. The risk of infertility associated with cancer treatment depends primarily on patient age and the type of therapy used; however, most female fertility preservation techniques, such as embryo or oocyte cryopreservation, ovarian tissue cryopreservation, ovarian transposition, and hormonal interventions, are not feasible during pregnancy and are more appropriately offered after delivery, together with assessment of reproductive function [33]. Concurrently, timely psychosocial support is essential to address the emotional concerns associated with cancer during pregnancy, a condition that intensifies psychological distress and increases the risk of anxiety, depression, and stress, underscoring the need for coping strategies and mental health support throughout follow-up [2]. Shared decision-making between the patient and a multidisciplinary team is fundamental to ensure that treatment plans align with the patient’s values and priorities.

**Figure 3:**
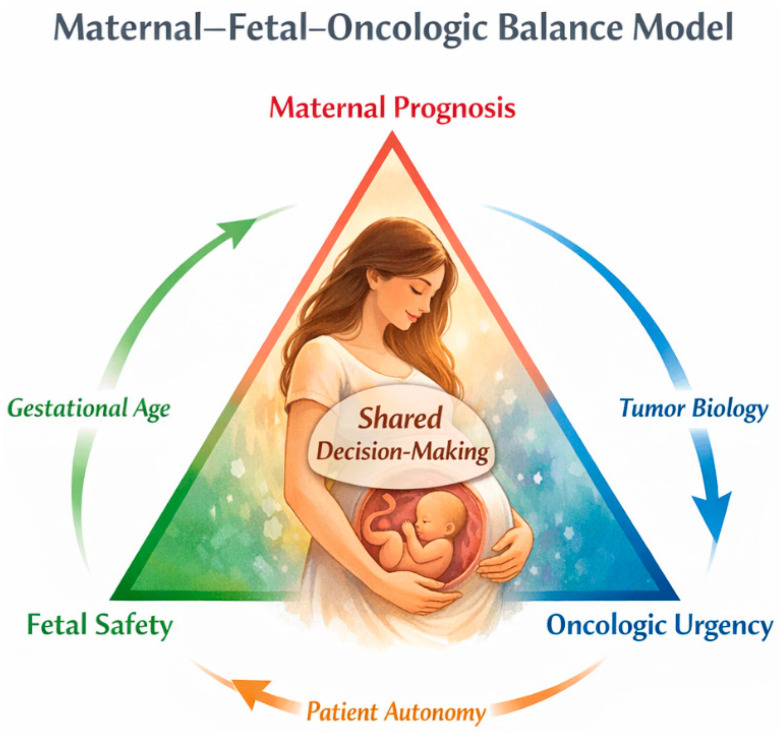
Multidisciplinary decision-making pathway for cancer diagnosed during pregnancy. Abbreviation: w, weeks of gestation.

To synthesize the ethical and clinical tensions inherent to cancer during pregnancy, Figure 3 illustrates the dynamic balance between maternal prognosis, fetal safety, and oncologic urgency. Rather than representing competing priorities, these domains interact continuously and are modulated by gestational age, tumor biology, and patient autonomy. Shared decision-making occupies the center of this framework, emphasizing that therapeutic choices must be individualized, transparent, and aligned with the values and goals of the pregnant woman.

## 10. Future Preservation

Fertility preservation represents a major concern for young women diagnosed with cancer during pregnancy. Most established fertility preservation strategies, such as oocyte cryopreservation, embryo cryopreservation, and ovarian tissue cryopreservation, cannot be performed during pregnancy due to the need for ovarian stimulation or surgical intervention. Consequently, these options are typically considered after delivery, once maternal oncologic treatment and reproductive potential have been reassessed [2,4].

Early counseling is therefore essential to inform patients about future reproductive options and potential risks of treatment-related infertility. Multidisciplinary collaboration between oncologists, reproductive specialists, and maternal–fetal medicine teams plays a key role in planning fertility-preserving strategies whenever clinically feasible [9].

## 11. Long-Term Outcomes

Overall, maternal outcomes in women diagnosed with cancer during pregnancy vary according to tumor type and disease stage at diagnosis. However, aggregated analyses indicate that these outcomes are largely comparable to those observed in non-pregnant women with cancer, with unfavorable prognosis primarily attributed to delayed diagnosis rather than to pregnancy itself [33]. Data from the International Network on Cancer, Infertility and Pregnancy (INCIP) registry, which includes 2359 women diagnosed between 2000 and 2022, support this heterogeneity by demonstrating an overall maternal mortality rate of 5.6%, with higher mortality associated with malignancies of poorer prognosis, such as lung cancer, gastroesophageal cancer, and acute leukemia. In these cases, maternal mortality was associated with adverse perinatal outcomes, including lower live birth rates, higher frequencies of elective cesarean delivery, and lower gestational age at delivery, resulting in increased prematurity [62].

Regarding long-term fetal and neonatal outcomes, available evidence suggests that intrauterine exposure to chemotherapy administered after the first trimester is not associated with significant impairments in postnatal growth or clinically relevant cognitive, neurological, or cardiac dysfunction. Systematic reviews and primary studies consistently indicate that cognitive development and school performance in these children generally fall within normal ranges [4,8,34,36]. Nevertheless, some follow-up studies have identified subtle alterations, such as mild reductions in verbal IQ scores, long-term visuospatial memory, and emotional regulation, without significant functional consequences [4,36]. Potential late complications, including neurodevelopmental alterations, cardiotoxicity, ototoxicity, endocrine disorders, and secondary malignancies, have been described, although without conclusive evidence of a direct causal association with intrauterine chemotherapy exposure [4,9].

Prematurity consistently emerges as the primary determinant of adverse neonatal outcomes and subsequent neurocognitive alterations, irrespective of exposure to maternal oncologic treatment, reinforcing the importance of avoiding elective preterm delivery whenever possible [8,34,36]. With respect to specific pharmacologic exposures, evidence suggests an increased risk of hearing loss in children exposed to cisplatin, justifying targeted auditory surveillance strategies, whereas available data on cardiotoxicity following prenatal exposure to anthracyclines are generally reassuring, showing only clinically insignificant echocardiographic differences compared with unexposed children [4,8].

Finally, interpretation of the available data must be undertaken with caution considering the methodological limitations inherent to published studies. The absence of control groups, use of non-standardized assessments, incomplete outcome reporting, risk of selection and observation bias, predominance of small sample sizes, and heterogeneity of therapeutic regimens limit statistical power and generalizability. Moreover, exclusion of pregnancies terminated due to concerns about fetal exposure and the influence of psychosocial factors related to the family context of parental cancer may contribute to underestimation of subtle or late effects [4].

## 12. Current Guidelines and International Recommendations

Current guidelines from international societies, including the American Society of Clinical Oncology, the European Society for Medical Oncology, and the European Society of Gynecological Oncology, recommend that care for pregnant women with cancer be supported by a multidisciplinary team and emphasize individualized decision-making. It is established that most diagnostic procedures can be safely performed during pregnancy, with preference given to ultrasonography and magnetic resonance imaging [4,28]. Although international societies such as ASCO, ESMO, and ESGO provide important guidance, differences in interpretation and local resources may influence clinical practice. Understanding areas of concordance among these guidelines can help clinicians navigate complex therapeutic decisions and improve the consistency of care.”

With respect to therapeutic management, there is consensus that surgical treatment is safe in any trimester. In addition, chemotherapy should be avoided during the first trimester, and radiotherapy is generally avoided overall. There is also agreement that delivery should be planned after 37 weeks of gestation, with an interval of 2–4 weeks between the last chemotherapy dose and delivery [4,28,63].

Conversely, several issues remain controversial. These include the safety of targeted therapies and immunotherapies due to the lack of robust studies, management of pregnant patients with rare malignancies or advanced-stage disease, and determination of the optimal timing of delivery in scenarios involving disease progression or risk of prematurity. These controversies arise primarily from the absence of prospective randomized studies [4,28,63].

To provide a structured comparative perspective, the main areas of convergence and divergence among these international recommendations are summarized in Table 4. While substantial agreement exists regarding diagnostic safety, trimester-based chemotherapy administration, surgical feasibility, and the avoidance of iatrogenic prematurity, important differences emerge in the interpretation of limited evidence, particularly concerning targeted therapies, immune checkpoint inhibitors, and complex advanced-stage scenarios. This comparative synthesis highlights both the maturity of current consensus in core management principles and the persistent gaps that continue to shape clinical uncertainty.

## 13. Future Directions and Research Gaps

Advances in the care of women with cancer during pregnancy depend decisively on the expansion, integration, and standardization of international prospective registries, such as the International Network on Cancer, Infertility and Pregnancy (INCIP) and the Cancer and Pregnancy Registry. These consortia currently represent the main sources of evidence for this population, enabling more robust multicenter analyses, comparisons across healthcare systems, and improved characterization of maternal, fetal, and neonatal outcomes over time. Strengthening these registries, together with harmonization of clinical definitions, therapeutic regimens, and evaluated outcomes, is essential to reduce data heterogeneity and enhance clinical applicability.

A critical unresolved gap concerns pregnancy-specific pharmacokinetics and pharmacodynamics. The profound physiological changes in pregnancy—including plasma volume expansion, increased renal clearance, altered protein binding, and hepatic metabolism—may significantly modify maternal and fetal exposure to antineoplastic therapies. However, most current recommendations are extrapolated from non-pregnant populations, limiting therapeutic precision and potentially resulting in both maternal undertreatment and unnecessary fetal exposure.

The absence of randomized clinical trials, for ethical and logistical reasons, remains the principal barrier to knowledge advancement. Consequently, major uncertainties persist regarding the long-term effects of intrauterine exposure to chemotherapy, as well as the safety of targeted therapies, immunotherapies, and novel agents, whose use in non-pregnant women has expanded rapidly. Furthermore, available data on late outcomes, including neurodevelopment, cardiac function, future fertility, and risk of secondary malignancies, remain limited, heterogeneous, and largely derived from small cohorts or incomplete follow-up.

Fertility preservation represents another central axis of investigation. Although infertility is a recurrent concern among young women undergoing oncologic treatment, available options during pregnancy are extremely limited. Thus, strategies for assessment and preservation of ovarian reserve are typically deferred to the postpartum period, when methods such as oocyte or embryo cryopreservation, ovarian tissue cryopreservation, and ovarian transposition may be considered. Future studies should explore not only the effectiveness of these approaches after pregnancy-associated cancer, but also their long-term psychosocial and reproductive impact.

Beyond the expansion of international registries, several priorities for future research should be emphasized. The development of pharmacokinetic biomarkers to tailor chemotherapy dosing during pregnancy, potentially using micro-sampling approaches, represents an important research priority. The creation of international consortia to standardize data collection on children exposed in utero to novel therapies, including immunotherapies and targeted agents, is also essential to assess their long-term safety. In addition, the integration of telemedicine and coordinated care models may improve access to multidisciplinary expertise for patients living far from specialized centers.

Ultimately, the future of cancer management during pregnancy lies in the consolidation of integrated care models, strengthening of international collaborative research, and incorporation of patient- and child-centered outcomes. Generation of higher-quality evidence is essential to transform decisions currently based on consensus and clinical experience into progressively more precise, safe, and individualized recommendations.

This review has several strengths. It provides a comprehensive synthesis of the current evidence on cancer during pregnancy, integrating data from clinical studies, international guidelines, and collaborative registries to present a multidisciplinary perspective on diagnosis, treatment, and obstetric management. However, some limitations should be acknowledged. As a narrative review, the selection and interpretation of the literature may be influenced by the authors’ judgment, and the evidence available in this field remains largely based on observational studies, registry-based analyses, and case reports. In addition, robust prospective data, particularly regarding emerging therapies and long-term offspring outcomes, remain limited, which should be considered when interpreting current recommendations.

## 14. Conclusions

Cancer during pregnancy, although uncommon, represents one of the most complex scenarios in contemporary medical practice, requiring careful integration of oncologic, obstetric, ethical, and reproductive principles. Available evidence supports that, in most cases, oncologic treatment can be delivered effectively without pregnancy termination and without compromising maternal or fetal outcomes, provided that care is based on individualized planning, appropriate gestational timing, and management by an experienced multidisciplinary team. Maternal prognosis is consistently more strongly related to tumor stage and disease biology than to pregnancy itself.

Despite significant advances over recent decades—particularly in the safe use of chemotherapy from the second trimester onward, refinement of surgical strategies, and obstetric management aimed at preventing iatrogenic prematurity—important gaps remain that limit high-level evidence-based practice. These gaps stem mainly from the unavoidable absence of randomized clinical trials, methodological heterogeneity of available observational studies, and the paucity of robust data on targeted therapies, immunotherapies, and novel agents during pregnancy, as well as on the late effects of intrauterine exposure.

In this context, decision-making must remain anchored in solid ethical principles, transparent communication, and shared decision-making, respecting the values, expectations, and priorities of the pregnant woman. Consolidation and expansion of international prospective registries, together with long-term follow-up of exposed mothers and children, represent the most promising pathway toward progressively more consistent evidence. Thus, care for women with cancer during pregnancy should be understood not as an insoluble dilemma, but as a continuously evolving field in which scientific advancement, international collaboration, and patient-centered care are fundamental to optimizing maternal and fetal outcomes.

## Figures and Tables

**Table 1:** Physiological adaptations of pregnancy with implications for cancer diagnosis and oncologic treatment.

Physiological System	Pregnancy-Related Change	Clinical/Oncologic Implication	Potential Diagnostic or Therapeutic Pitfall
Cardiovascular	↑ Plasma volume; ↑ cardiac output; ↓ systemic vascular resistance	Hemodilution of drugs; altered pharmacokinetics; masking of hypovolemia	Delayed recognition of shock; underestimation of disease severity
Renal	↑ Glomerular filtration rate	Increased clearance of chemotherapeutic agents and supportive drugs	Subtherapeutic drug exposure if underdosed
Hematologic	Physiological anemia; hypercoagulable state	Baseline anemia may mimic malignancy-related cytopenias; ↑ thrombotic risk	Misinterpretation of laboratory abnormalities; underuse of thromboprophylaxis
Gastrointestinal	Nausea, vomiting, reflux, constipation	Symptom overlap with malignancy or treatment-related toxicity	Delayed cancer diagnosis; attribution of red flags to pregnancy
Immune	Shift toward immune tolerance (↑ Treg, Th2 predominance)	Potential reduced tumor immune surveillance; interference with immunotherapy mechanisms	Increased risk of immune-related fetal or placental effects with ICIs
Breast	Glandular hypertrophy, increased density, mastalgia	Reduced sensitivity of physical examination and imaging	Delayed diagnosis of breast cancer; false reassurance

Abbreviations: Treg, regulatory T cells; Th2, T helper type 2 cells; ICIs, immune checkpoint inhibitors; ↑, increased; ↓, reduction.

**Table 2:** Safety of anticancer therapies according to gestational age.

Therapeutic Modality	First Trimester	Second Trimester	Third Trimester	Key Considerations
Surgery	Generally avoided if elective	Preferred timing	Feasible with precautions	Multidisciplinary planning; fetal monitoring after viability
Chemotherapy (cytotoxic)	Contraindicated (high teratogenic risk)	Generally safe	Generally safe; stop at 34–35 weeks	Dose based on actual body weight; avoid near delivery
Radiotherapy	Contraindicated	Avoided; rare exceptions	Rarely feasible in selected supradiaphragmatic tumors	Fetal dose should remain <100 mGy; strict dosimetry required
Targeted therapy (anti-HER2, TKIs)	Contraindicated	Contraindicated	Contraindicated	Associated with oligohydramnios, fetal toxicity, and neonatal complications
Immunotherapy (ICIs)	Contraindicated	Contraindicated	Contraindicated	Risk of miscarriage, placental insufficiency, fetal immune dysregulation
Supportive care (antiemetics, G-CSF)	Selective use	Generally safe	Generally safe	Ondansetron: caution in 1st trimester; individualized risk–benefit

Abbreviations: anti-HER2, anti-human epidermal growth factor receptor 2; TKIs, tyrosine kinase inhibitors; ICIs, immune checkpoint inhibitors; G-CSF, granulocyte colony-stimulating factor; mGy, milligray.

**Table 3:** Cancer-specific considerations for diagnosis and management during pregnancy.

Cancer Type	Diagnostic Challenges	Preferred Treatment During Pregnancy	Obstetric Considerations	Placental/Fetal Metastasis Risk
Breast cancer	Dense breast tissue; symptom overlap	Surgery any trimester; chemotherapy after 1st trimester	Avoid iatrogenic prematurity; vaginal delivery preferred	Moderate (≈12% placental involvement)
Cervical cancer	Limited use of CT/PET; bleeding risk with biopsy	Conization or simple trachelectomy (early stages); NACT if advanced	Cesarean delivery often indicated in invasive disease	Low
Ovarian cancer	Adnexal masses common in pregnancy	Conservative surgery; NACT in advanced disease	Risk of preterm delivery; close fetal surveillance	Low
Hematologic malignancies	Symptoms mimic pregnancy (fatigue, dyspnea)	Chemotherapy after 1st trimester (regimen-specific)	High prematurity risk if treatment delayed	Rare
Melanoma	Delay in excision; nodal assessment challenges	Surgical excision; SLNB with technetium	Vaginal delivery acceptable	High (≈30%)
Thyroid cancer	Physiologic thyroid changes	Surgery in 2nd trimester or postpartum	Usually, minimal obstetric impact	Minimal
Gestational choriocarcinoma	Rare; may present postpartum	Chemotherapy (highly chemosensitive)	Intensive maternal–fetal monitoring	Very high (fetoplacental origin)

Abbreviations: CT/PET, computed tomography/positron emission tomography; NACT, neoadjuvant chemotherapy; SLNB, sentinel lymph node biopsy.

**Table 4:** Areas of Consensus and Ongoing Controversy in International Guidelines on Cancer During Pregnancy (ASCO, ESMO, ESGO).

Clinical Aspect	ESGO	ASCO	ESMO
General principle	Multidisciplinary tumor board recommended; structured coordination in gynecologic oncology	Multidisciplinary management in experienced centers; maternal prognosis prioritized	Referral to specialized centers; registry-based evidence emphasized
Timing of chemotherapy	Similar trimester-based approach; treatment aligned with gestational age	Contraindicated in 1st trimester; may be administered after 14 weeks	Avoid during organogenesis; may start in 2nd/3rd trimester
Targeted therapy (anti-HER2)	Contraindicated during pregnancy; limited case data acknowledged	Insufficient safety data; generally contraindicated due to fetal risk	Contraindicated; risk of oligohydramnios highlighted
Immunotherapy (ICIs)	Not recommended outside exceptional circumstances	Limited evidence; generally discouraged during pregnancy	Insufficient safety data; potential immune-mediated fetal risks discussed
Radiotherapy	Strong emphasis on minimizing fetal exposure; individualized risk assessment	Generally avoided; may be considered in highly selected cases with strict fetal dose constraints	Avoided when possible; case-by-case evaluation with shielding and fetal dose assessment
Surgical management	Recommends adherence to standard oncologic principles with obstetric coordination	Considered safe in all trimesters with obstetric precautions	Surgery feasible during pregnancy; laparoscopy acceptable in selected cases
Sentinel lymph node biopsy	Radiocolloid preferred; blue dye generally discouraged	Radiocolloid permitted; blue dye discouraged	Radiocolloid acceptable; avoid isosulfan blue
Delivery timing	Recommends avoiding preterm delivery solely for maternal treatment	Avoid iatrogenic prematurity when oncologically feasible	Emphasis on fetal maturity and continuation of pregnancy when safe
Long-term offspring follow-up	Structured pediatric follow-up recommended	Encouraged; available data generally reassuring	Registry-based follow-up supported
Ethical framework	Multidisciplinary ethical deliberation when maternal and fetal interests may diverge	Shared decision-making and informed maternal autonomy emphasized	Maternal prognosis central; individualized counseling recommended

Abbreviations: anti-HER2, anti-Human Epidermal Growth Factor Receptor 2 therapy; ESGO, European Society of Gynaecological Oncology; ASCO, American Society of Clinical Oncology; ESMO, European Society for Medical Oncology.

## Data Availability

No new data were created or analyzed in this study. Data sharing is not applicable to this article.
